# Inter-brain synchrony to delineate the social impairment in autism spectrum disorder: a systematic review on hyperscanning studies

**DOI:** 10.1093/psyrad/kkaf025

**Published:** 2025-09-18

**Authors:** Yuhang Li, Shuo Guan, Dalin Yang, Dongyun Li, Qiong Xu, Yingchun Zhang, Rihui Li

**Affiliations:** Centre for Cognitive and Brain Sciences, Institute of Collaborative Innovation, University of Macau, Macau S.A.R. 999078, China; Department of Psychology, Faculty of Social Sciences, University of Macau, Macau S.A.R. 999078, China; Centre for Cognitive and Brain Sciences, Institute of Collaborative Innovation, University of Macau, Macau S.A.R. 999078, China; Department of Psychology, Faculty of Social Sciences, University of Macau, Macau S.A.R. 999078, China; Mallinckrodt Institute of Radiology, Washington University School of Medicine, St. Louis, MO 63110, USA; Department of Child Health Care, Children's Hospital of Fudan University, Shanghai 201102, China; Department of Child Health Care, Children's Hospital of Fudan University, Shanghai 201102, China; Department of Biomedical Engineering, University of Miami, Coral Gables, FL 33146, USA; Centre for Cognitive and Brain Sciences, Institute of Collaborative Innovation, University of Macau, Macau S.A.R. 999078, China; Department of Electrical and Computer Engineering, Faculty of Sciences and Technology, University of Macau, Macau S.A.R. 999078, China

**Keywords:** autism spectrum disorder, social impairment, inter-brain synchrony, hyperscanning, brain imaging

## Abstract

Autism spectrum disorder (ASD) is a common neurodevelopmental disorder marked by significant deficits in social interaction and restricted repetitive behaviors. Despite rigorous research efforts, the early and effective diagnosis and intervention of ASD remain challenging, due primarily to its considerable heterogeneity and complex neurobiological underpinnings. Traditional neuroimaging techniques have largely focused on individual brain responses to social stimuli, often overlooking the critical interactive dynamics that contribute to social impairments in individuals with ASD. This review explored hyperscanning, an innovative neuroimaging approach that features simultaneous recording of brain activity across multiple individuals, to enhance our understanding of the neural mechanisms underlying social difficulties in ASD. By searching published articles conducted between 2000 and 2024, we found eight empirical studies conducted between 2012 and 2024, which employed various brain imaging techniques. We analyzed and summarized participant demographics, experimental designs, and key outcomes, with a particular focus on inter-brain synchrony (IBS) as a measure of social engagement and the quality of interpersonal interactions. Our review identified specific patterns of neural synchrony that correlate with the severity of ASD symptoms. Furthermore, we critically evaluated the limitations of current studies and proposed future research directions, highlighting the need for more nuanced hyperscanning methodologies. Such advancements could significantly deepen our understanding of social impairments in ASD and inform targeted intervention strategies. This comprehensive review aimed to assess the potential of hyperscanning techniques to propel progress in ASD research and intervention, ultimately contributing to more effective clinical practices.

## Introduction

Autism spectrum disorder (ASD) is a common but complex neurodevelopmental disorder characterized by impairments in social interaction and restricted repetitive behaviors (Lord *et al*., 2020). The prevalence of ASD is approximately 1 in 100 globally (Zeidan *et al*., 2022) and 1 in 142 in China (Zhou *et al*., 2020). The *Diagnostic and Statistical Manual of Mental Disorders*, Fifth Edition (DSM-5) criteria for ASD diagnosis include two core symptoms: (i) difficulties in communication and interaction, and (ii) restricted interests and repetitive behaviors (American Psychiatric Association & American Psychiatric Association, 2013). Challenges in social communication can manifest as difficulties in understanding social cues (Jellema *et al*., 2009), maintaining eye contact (Senju and Johnson, 2009), and interpreting nonverbal communication (G. K. Chen *et al*., 2006; Chiang *et al*., 2008).

The increasing prevalence of ASD, in combination with the lack of social adaptiveness and the need for life-long support for individuals with ASD, brings a great amount of economic and educational costs for families and governments every year (Rogge and Janssen, 2019). However, despite its substantially negative impact, the accurate diagnosis and effective intervention of ASD can be difficult, especially in its early stages. This can be attributed to the high heterogeneity of ASD, which poses a significant challenge in unraveling the complex mechanisms underlying ASD, particularly in understanding the biological basis of its core phenotypes such as anxiety and avoidance during social interaction (Guo *et al*., 2022). Gaining more insight into the social impairment in ASD individuals is expected to identify objective neural biomarkers for the assessment and monitoring of ASD, promoting early intervention and effective treatment of the disease.

Advanced brain imaging techniques such as functional magnetic resonance imaging (fMRI), functional near-infrared spectroscopy (fNIRS), magnetoencephalography (MEG), and electroencephalography (EEG) have enabled researchers to identify the impaired brain networks underlying different levels of ASD social impairment, from resting state to inferring the mental state of others, mostly by displaying manipulated social stimuli on the screen in the laboratory (Kleinhans *et al*., 2011; Duan and Chen, 2022; B. Chen *et al*., 2023; Lau *et al*., 2024; Wan *et al*., 2024; Zhu *et al*., 2024). However, most studies have exclusively examined single participants' brain responses to socially relevant pictures or video clips displayed on a monitor, which might fail to reflect the core symptom of ASD, i.e. neural dysfunction occurring in natural social interaction. There remain important challenges in understanding the neurobiological underpinnings that are representatively related to the social impairment in children with ASD.

**Figure 1:**
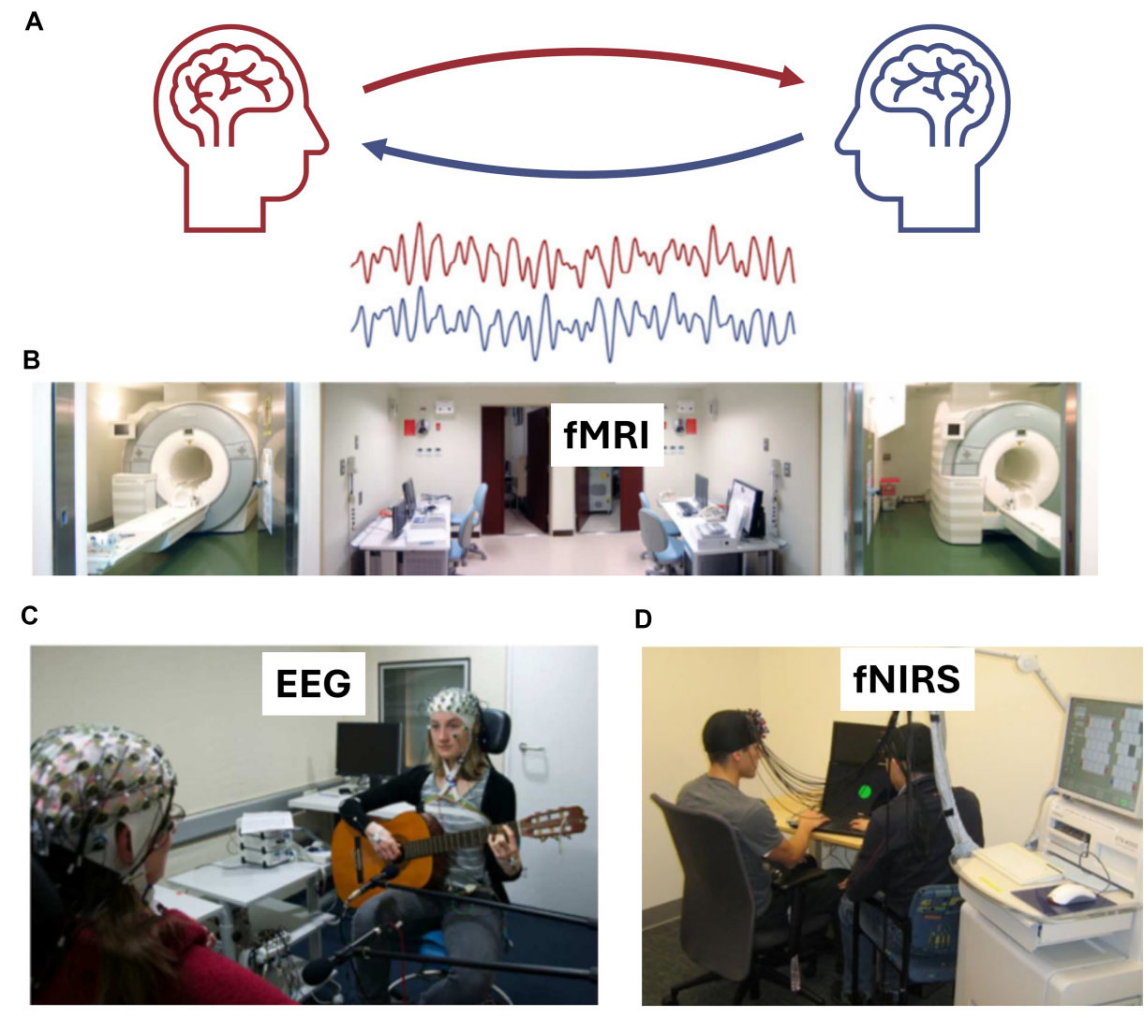
Hyperscanning setups with different modalities. (**A**) Conceptual demonstration of inter-brain synchrony measured by hyperscanning. (**B**) Functional magnetic resonance imaging (fMRI)-based hyperscanning (adapted from Koike *et al*., 2019). (**C**) Electroencephalography (EEG)-based hyperscanning (adapted from Acquadro *et al*., 2016). (**D**) Functional near-infrared spectroscopy (fNIRS)-based hyperscanning (adapted from Cui *et al*., 2012).

In recent years, a technique known as hyperscanning, which allows for the simultaneous recording of brain activity from multiple individuals, has gained significant attention in social cognitive neuroscience (Li *et al*., 2021; Kelsen *et al*., 2022; Hakim *et al*., 2023; Pan *et al*., 2023; Zhao *et al*., 2024). Using different brain imaging techniques (Fig. 1B–D), hyperscanning provides a unique opportunity to investigate how multiple brains communicate, termed inter-brain synchrony (IBS) (Fig. 1A), within a more ecologically valid and interactive context. Numerous lines of evidence have demonstrated that the IBS between two individuals can reflect their engagement and relationship during naturalistic social interaction (Cui *et al*., 2012; Hu *et al*., 2017; Pan *et al*., 2017, 2018; Li *et al*., 2021;). Additionally, previous studies have found associations between IBS of parent–child interactions and children's behavior (Quiñones-Camacho *et al*., 2020; Reindl *et al*., 2018). The IBS observed in hyperscanning studies appears to be affected by the quality of interaction between participants, which opens up new avenues for understanding the neural mechanism underlying social impairments in individuals with ASD during real-life social interactions. In this context, studies have attempted to explore the neural dynamics linked to social impairment in children with ASD using different hyperscanning settings (H. Tanabe *et al*., 2012; Wang *et al*., 2020 Quiñones-Camacho *et al*., 2021; Kruppa *et al*., 2021; Hirsch *et al*., 2022; Key *et al*., 2022; Du *et al*., 2024). Yet, there is no comprehensive summary in terms of the key considerations for applying hyperscanning techniques in social interaction for children with ASD.

To bridge this gap, this article systematically reviewed the dyads' characteristics, paradigms, and hyperscanning settings and the key findings of those studies that applied hyperscanning techniques to children with ASD. The structure of the review is as follows. First, we introduce the advantage of hyperscanning techniques in exploring the neural mechanisms underlying the social impairment of ASD. Second, we describe the criteria and procedure of the literature review. Then, we described the methods, study design, and key findings of the included literature from multiple perspectives. Finally, we critically assess the limitations encountered in existing hyperscanning studies focused on ASD and offer insights into potential future directions for research.

## Method

**Figure 2:**
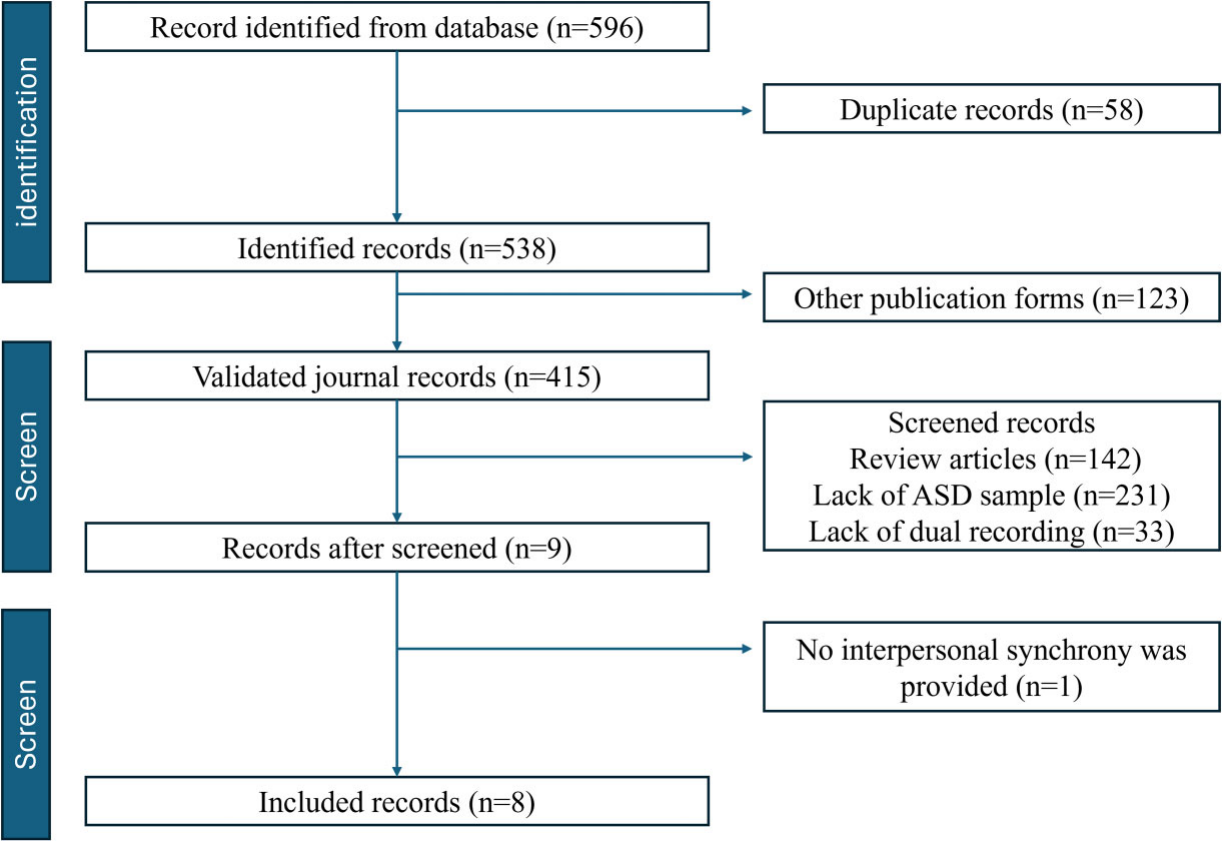
The PRISMA flowchart of the literature search and screening strategy (Page *et al*., 2021).

We conducted this review following the Preferred Reporting Items for Systematic Reviews and Meta-Analyses (PRISMA) guidelines (Page *et al*., 2021). We searched articles published between 2000 and 2024 from the major databases of academic publications, including PubMed, Web of Science, ProQuest, and ScienceDirect. The following keywords were used to search the articles: (“Autism” OR “pervasive developmental disorder ” OR “Asperger Syndrome”) AND (“hyperscanning” OR “inter-brain synchrony” OR “inter-brain synchronization” OR “inter-brain coupling” OR “interpersonal neural synchrony” OR “interpersonal neural synchronization” OR “interpersonal neural coupling” OR “brain-to-brain coupling”). The entire selection process is depicted in Fig. 2.

We only included empirical studies that met the following criteria: (i) recruited dyads of participants and recorded their brain signals simultaneously using brain imaging techniques such as fNIRS, EEG, MEG, and fMRI; (ii) included “ASD dyads” with at least one individual diagnosed with ASD; and (iii) engaged the dyads in real-time social interaction during the brain recording.

After excluding duplicate records, we obtained 538 records of the search results; 123 are books, conference abstracts, or theses. We then screened the articles based on their titles, summaries, and abstracts. The remaining eight studies were further checked based on their full-text contents. This search strategy resulted in eight key articles published between 2012 and 2024. For each study included, we extracted the following information: author(s), year of publication, participant characteristics (diagnosis, age, sex ratio, and sample size), cognitive-behavioral measurement, and main findings.

## Results

While the study design and results of the selected publications vary significantly, we have attempted to summarize the key information of these studies into several components, including the characteristics of participants, experimental paradigms, hyperscanning modalities, and main findings. These components are summarized in Table 1 and elaborated as follows.

**Table 1:** Summary of key characteristics and findings of the reviewed articles.

	Dyads characteristic			Brain scan parameters		Tasks and assessments		
	Age (years)	Partner	ASD/TD (male:female)	Channels	Brain coverage	Paradigm	Scale	Key findings
Du *et al*., 2024	6.1 ± 1.0/5.1 ± 1.3	Experimenter	15:5/16:4	20 channels fNIRS	Bilateral IFG, IPL, and TPJ	Movement imitation	SRS; ABC; PPVT-R; CRT	Significant lower IBS of ASD group than TD group at IPL
Tang *et al*., 2023	8.0 ± 2.2/8.2 ± 2.1	Parents	30:6/21:14	22 channels fNIRS	Right frontal and right temporal regions	Computer-based cooperation	ADOS-2	No significant differences between the ASD and TD groups in IBS during cooperation task
Key *et al*., 2022	12.6 ± 2.2/NO TD	Experimenter	17:17/NO TD	128 channel EEG	Full brain	Conversation	SCQ; NEPSY Theory of Mind (TOM) subtests; ADOS-2; WASI	Social interaction induced IBS positively correlated with social affect and symptom severity of individuals with ASD
Hirsch *et al*., 2022	25.0 ± 4.9/26.0 ± 5.8	Sender-matched experiment partner	14:3/19	54 channels fNIRS	Full brain	Eye contact	ADOS-2; WASI-II, AQ; BAPQ; SRS; BAI; STAI; LSAS	IBS at TPJ between ASD adults and experimenter was reduced compared with that between TD and experimenter
Quiñones-Camacho *et al*., 2021	25.1 ± 6.2/26.3 ± 7.7	Experimenter	24:0/26:0	8 channels; fNIRS	PFC & TPJ	Conversation	SRS; ADOS-2	IBS at TPJ negatively correlated with ASD social communication scores
Kruppa *et al*., 2021	13.5 ± 3.0/13.5 ± 3.0	Parents/stranger	18:0/41	22 channels; fNIRS	Forehead frontal cortices	Computer-based cooperation and competition	SRS; SCQ; FBB-HKS (a German ADHD rating scale)	No evidence of reduced IBS between ASD children and parents/stranger; No evidence of ASD symptom-related IBS
Wang *et al*., 2020	8.2 ± 1.7/NO TD	Parents	15:1/NO TD	22 channels NIRS	Forehead frontal cortices	Computer-based cooperation	AQ; SRS;	IBS at frontal region between ASD children and parents was correlated with social difficulty of ASD children
Tanabe *et al*., 2012	25.1 ± 5.3/23.8 ± 3.5	TD partner	16:5/19	1.5T 3T fMRI	Full brain	Joint attention task	AQ; WAIS-III; DISCO	IBS at IFG between ASD adults and TD fellows was reduced compared with that between TD dyads.

SRS: Social Responsiveness Scale; ABC: Autism behavior checklist; PPVT-R: Peabody Picture Vocabulary Test - Revised; CRT: Combined Raven’s Test; ADOS-2: Autism Diagnostic Observation Schedule, Second Edition; SCQ: Social Communication Questionnaire; WASI: Wechsler Abbreviated Scale of Intelligence; AQ: Autism Quotient; BAPQ: Broad Autism Phenotype Questionnaire; BAI: Beck Anxiety Inventory; STAI: LSAS: Liebowitz Social Anxiety Scale; DISCO: Diagnostic Interview for Social and Communication Disorders.

### Characteristics of participated dyads

#### Age

Three of the studies recruited only adults with ASD (Tanabe *et al*., 2012; Quiñones-Camacho *et al*., 2021; Hirsch *et al*., 2022) meanwhile, two studies recruited adolescents with ASD aged between 8 and 18 years (Key *et al*., 2022; Kruppa *et al*., 2021). Three studies recruited young children with ASD with ages between 5 and 11 years (Wang *et al*., 2020; Tang *et al*., 2023; Du *et al*., 2024). Overall, the age range in the existing studies is relatively large.

#### Gender

Among the eight selected studies, only one study recruited an equal number of male and female participants with ASD and compared differences in the IBS between the genders (Key *et al*., 2022). Two studies focused exclusively on male participants with ASD as their subjects of investigation (Kruppa *et al*., 2021; Quiñones-Camacho *et al*., 2021). In total, the male-to-female ratio of individuals with ASD across these studies was 171:43, similar to the gender ratio in the ASD population.

#### Screen criteria

The main screen criteria for ASD samples across the included studies are (i) professional ASD diagnosis and (ii) normal-level IQ. All selected studies required that individuals with ASD should be diagnosed by professional pediatricians in licensed hospitals according to the criteria of ASD in DSM-5 (American Psychiatric Association & American Psychiatric Association, 2013), except for one study carried out before the publication of DSM-5 (Tanabe *et al*., 2012). Most included studies recruited ASD individuals with at least a normal level IQ, except Tang *et al*. (2023), who noted that they recruited high-function ASD children without offering IQ, and Du *et al*. (2024), who recruited ASD children with significantly lower IQ than typical developing (TD) children.

#### Dyads

The configurations of the dyads varied among the included hyperscanning studies. Each type of dyad configuration creates a distinctive social interaction environment during the task for children with ASD, probing different aspects of the social impairments of ASD. Wang *et al*. (2020) recruited parent–autistic child dyads. As the first significant other of children, parents play a unique and essential role in the development of children’s social ability (Lamb and Lewis, 2011). The atypical parent–child interaction has been reported as a common social behavior characteristic of children with ASD (Freeman and Kasari, 2013; Jurek *et al*., 2023). Investigating the atypical IBS pattern in parent–autistic child dyads could help to reveal the children's difficulty in intimacy and relationship building. Similarly, Kruppa *et al*. (2021) recruited parent–ASD child dyads and also included stranger–ASD child dyads to examine the influence of relationships on IBS. The stranger–child dyads probed the social difficulty of children with ASD in a real-life environment.

Tang *et al*. (2023) paired parents of children with ASD into parent–parent dyads for comparison with parent–child dyads. They set the parent–parent group against the parent–child group to minimize the ASD–TD difference other than the ASD symptoms of children. However, this configuration also introduced dyads’ relationship (parent–child vs couples) as a confounder. Several studies further minimized the difference between ASD and TD groups by employing the same experimenters as partners for all ASD and TD participants (Du *et al*., 2024; Hirsch *et al*., 2022; Key *et al*., 2022; Quiñones-Camacho *et al*., 2021). These studies reduced the influence of interaction partners of children in the comparison between ASD and TD groups.

A special dyad setting was adopted in a study that recruited age- and gender-matched TD individuals as the interaction partners of individuals with ASD to investigate the social difficulty of ASD individuals during interaction with their peers (Tanabe *et al*., 2012). The social interaction between peers significantly influences the development of children's social abilities (Kelly *et al*., 2008). Peer-mediated intervention has been proposed as an effective method of ASD treatment (Chang and Locke, 2016). Probing the social difficulties between children with ASD and their peers could advance the current understanding of the peer relationship of children with ASD as intervention targets.

### Hyperscanning modality

As shown in Table 1, previous hyperscanning studies have utilized various brain imaging techniques, including fMRI, fNIRS, and EEG (Key *et al*., 2022). The earliest among those originated from hyperscanning studies based on fMRI. The authors employed fMRI devices of 1.5 and 3T, revealing the correlation of brain hemodynamic response time series between individuals with ASD and TD peers during social interactions (Tanabe *et al*., 2012). In recent years, hyperscanning studies on ASD have employed more portable fNIRS devices. The number of channels in these studies varied widely, from eight to 58 per person. All of them covered the frontal areas, and three of them also covered bilateral temporal regions (Hirsch *et al*., 2022; Quiñones-Camacho *et al*., 2021; Du *et al*., 2024).

### Hyperscanning tasks

**Figure 3:**
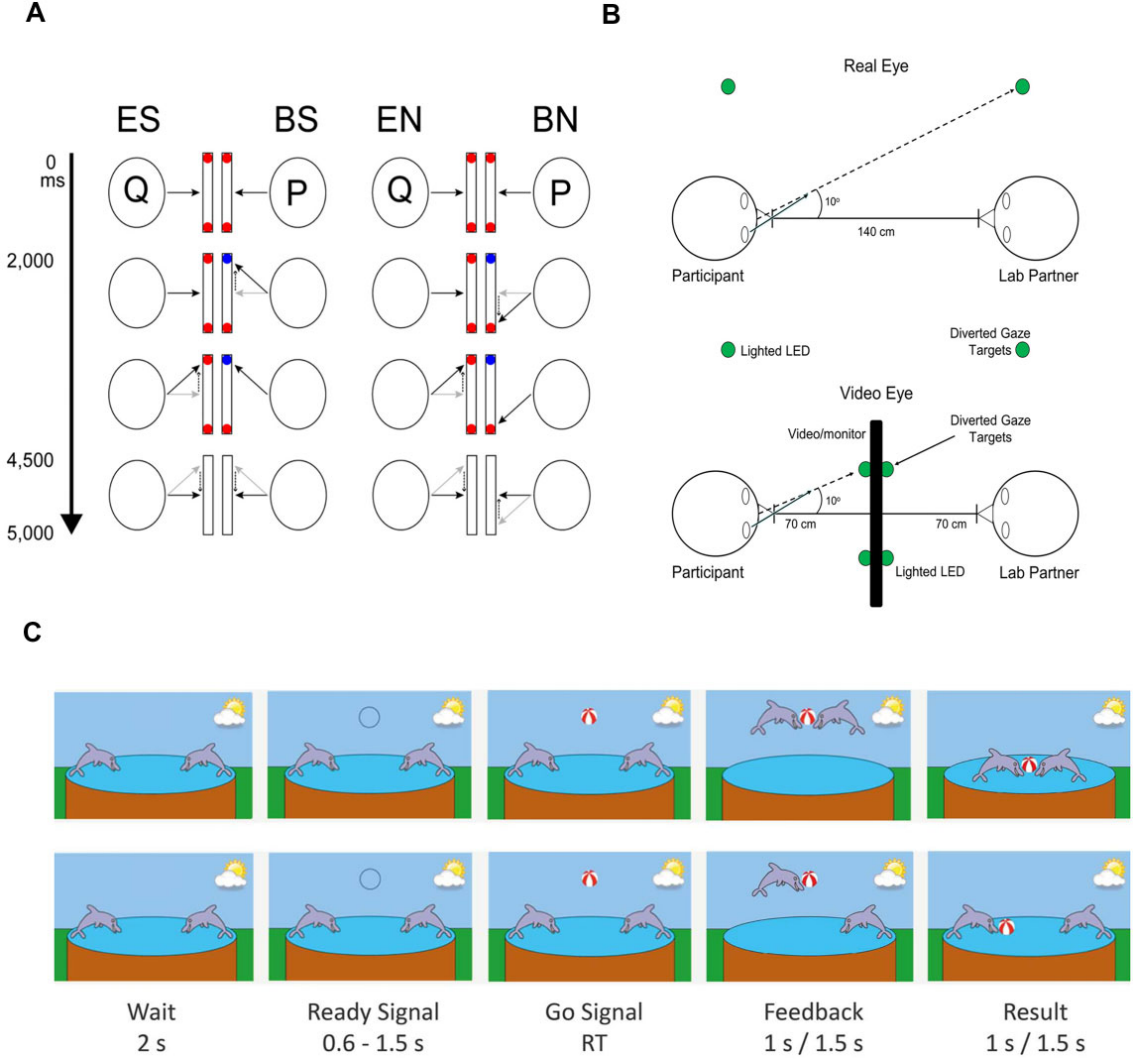
Major hyperscanning paradigms implemented in the reviewed studies. (**A**) Eye gaze-directed attention task (adapted from Tanabe *et al*., 2012). (**B**) Eye contact task (adapted from Hirsch *et al*., 2022). (**C**) Computer-based button press task (adapted from Kruppa *et al*., 2021).

The early application of the hyperscanning method was inspired by traditional joint gaze social cognition tasks. During a simultaneous fMRI recording, Tanabe *et al*. (2012) displayed two dots on the left and right side of the bottom half of the screen and projected the real-time video recording of the partner's eyes on the top of the screen (Fig. 3A). Participant A in dyads was directed to gaze at the left or right dots according to a color change cue, while Participant B was required to gaze at the dot on the same or opposite side according to Participant A's eye gaze movement. As two participants played the roles of A and B in turns, the experiment distinguished four types of trials: eye-cued directed vs ball-cued directed, and same side vs opposite side.

Similarly, Hirsch *et al*. (2022) applied hyperscanning fNIRS recording to ASD–TD dyads during eye contact (Fig. 3B). In the real eye contact condition, they required the participants to sit quietly face-to-face and look at each other's eyes. In the fake eye contact condition, the authors played videos of their partner's eye contact on the screen, simulating the real eye contact condition. This experimental setting validated the IBS during eye contact by precisely controlling the visual input between real eye contact and fake eye contact conditions.

Quiñones-Camacho *et al*. (2021) implemented the hyperscanning method in more naturalized experiments. The authors engaged ASD individuals in a free conversation with a trained female research assistant, who interacted with all participants. Their brain hemodynamic activities were recorded spontaneously during the whole conversation using fNIRS. The task consisted of four prompts, such as “Where would you go on vacation?”. The dyads talked for about 2 min for each prompt. Key *et al*. (2022) conducted a similar experiment using the EEG hyperscanning technique. Differently, they let the dyads talk about a fun day in the past or the future for 5 min. For naturalized experiments with children, Du *et al*. (2024) engaged children with ASD and an experimenter in a movement imitation task. The children with ASD were asked to replicate the movements synchronously while the experimenter showed them a series of gestures.

Three studies have utilized another computer-based cooperation task, a widely used paradigm for hyperscanning studies (Cui *et al*., 2012). Wang *et al*. (2020) engaged parents and children in a cooperation task and a single-person task. During the cooperation task, pairs of participants were required to press a button simultaneously after receiving a cue displayed on the screen, whereas, in the single-person task, they had to press the button within 1 s after the same cue. Kruppa *et al*. (2021) implemented the same task setting except that they substituted the single-person task with a competition task, during which the participants were required to press the button as soon as possible to win a prize (Fig. 3C). Using a similar paradigm, Tang *et al*. (2023) classified the strategy of parents into three groups: “Delayed response,” “Immediate response,” and “Not specific.” The “Delayed response” described the strategy in which the parents allowed a certain delay between seeing the signal and pressing keys. The “Immediate response” described the strategy that the parents pressed the button immediately after seeing the signal. The strategy could not be classified into these two categories and was coded as “Not specific.”

### Metrics for assessing inter-brain synchrony

In hyperscanning studies, the strength of coupling between two brains (i.e. IBS) can be assessed by a variety of metrics, including Pearson's correlation, wavelet transform coherence (WTC), or phase synchrony.

#### Pearson's correlation

Two articles employed traditional functional connectivity measurement, Pearson's correlation, as an indicator of IBS (Quiñones-Camacho *et al*., 2021; Tanabe *et al*., 2012). This approach captures the time-domain synchrony of two time series, omits the frequency-specific effects, and is suitable for estimating IBS from slow brain signals such as fMRI. As shown in Equation 1, the time series of two channels are represented by *X_i_, Y_i_* (i = 1, 2, 3,…, *n*), and their Pearson's correlation is denoted by *r* and given as:


(1) \begin{eqnarray*}
r = \frac{{\mathop \sum \nolimits_{i = 1}^{\ n} \left( {{{X}_i} - \overline{X}} \right)\ \left( {{{Y}_i} - \overline{Y}} \right)}}{{\sqrt {\mathop \sum \nolimits_{i = 1}^{\ n} \left( {{{X}_i} - \overline{X}} \right)} \sqrt {\mathop \sum \nolimits_{i = 1}^{\ n} \left( {{{Y}_i} - \overline{Y}} \right)} }}
\end{eqnarray*}

where $\bar{X}$ and $\bar{Y}\ $ represent the mean value of two time series *X_i_* and *Y_i_*, respectively.

#### Wavelet transform coherence

Three studies used WTC to estimate IBS between the dyads (Kruppa *et al*., 2021; Tang *et al*., 2023; Q. Wang *et al*., 2020; Du *et al*., 2024). Briefly, WTC is a method of analysis that measures the cross-correlation between two signals as a function of frequency and time (Grinsted *et al*., 2004). It calculates the localized coherence coefficient as a value between 0 and 1. Mapping this coherence value uncovers inter-signal phenomena that might not be discoverable through traditional time-series analysis (Cui *et al*., 2012; Léné *et al*., 2020). Because the time–frequency map derived from WTC allows researchers to focus on specific frequency bands and moments physiologically relevant for hemodynamic signals and social processes, it is considered the most suitable method for fNIRS-based hyperscanning studies since it was proposed by Cui *et al*. (2012). To account for the similarity of the frequency–time domain data between two channels, the wavelet coherence of time *n* and scale *s* was calculated as


(2) \begin{eqnarray*}
R_n^2\left( s \right) = \frac{{{{{\left| {S\left( {W_n^X\left( s \right)W_n^Y{{{\left( s \right)}}^*}} \right)} \right|}}^2}}}{{S\left( {{{{\left| {W_n^X\left( s \right)} \right|}}^2}} \right) \cdot S\left( {{{{\left| {W_n^Y\left( s \right)} \right|}}^2}} \right)}}
\end{eqnarray*}

where $W_n^X( s )$ and $W_n^Y( s )$ denote the continuous wavelet transform (CWT) of two time series *X* and *Y* at time *n* and scale *s*, * denotes complex conjugation, and *S* denotes a smoothing operator in time and scale.

#### Phase synchrony

Besides the synchrony measured by Pearson correlation and WTC, phase synchrony is also applied in ASD hyperscanning research (Key *et al*., 2022). This method is more suited to EEG and MEG data, because the high temporal resolution (milli second level) of EEG allows the phase dynamics of the signal to be better interrogated. Key *et al*. (2022 used the circular correlation coefficient (CCor) to estimate IBS. The CCor of time series (*k =* 1,…, *N*) of two channels *X* and *Y* is defined as


\begin{eqnarray*}
\textit{CCor}{{r}_{X,Y}} = \frac{{\mathop \sum \nolimits_{k = 1}^N {\mathrm{sin}}\,\left( {\phi _k^x - \overline {{{\phi }^x}} } \right)sin\left( {\phi _k^y - \overline {{{\phi }^y}} } \right)}}{{\sqrt {\mathop \sum \nolimits_{k = 1}^N {\mathrm{sin}}{{}^2}\left( {\phi _k^x - \overline {{{\phi }^x}} } \right)sin\left( {\phi _k^y - \overline {{{\phi }^y}} } \right)} }}
\end{eqnarray*}

where ϕ^*x*^ and ϕ^*y*^ represent the phases of the time series.

### The distinct IBS pattern of ASD dyads during social interaction

**Figure 4:**
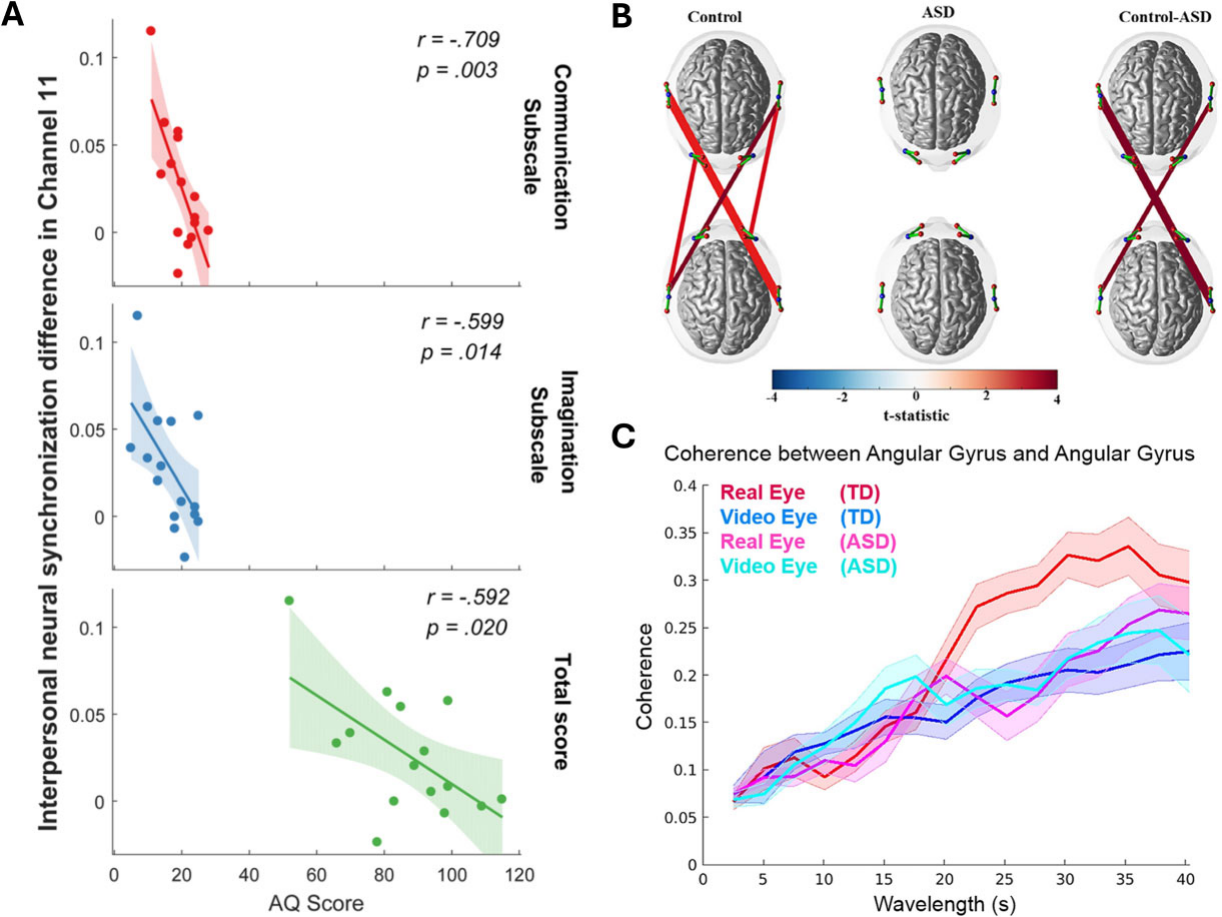
Major findings of ASD symptom-related IBS in the reviewed studies. (**A**) The IBS between children with ASD and their parents correlated with ASD symptom severity measured by the Autism-Spectrum Quotient (AQ) (adapted from Wang *et al*., 2020). (**B**) The IBS between ASD adults and experimenters was reduced compared to the TD control group (adapted from Quiñones-Camacho *et al*., 2021). (**C**) Lower eye-contact-evoked IBS at the TPJ was found between ASD adults and TD peers compared to the TD group (adapted from Hirsch *et al*., 2022).

Reduced IBS of ASD dyads compared with TD dyads was generally expected in hyperscanning studies, as ASD individuals are commonly characterized by their social interaction defect. Supporting this, Tanabe *et al*. (2012) found significantly reduced IBS of ASD–TD pairs compared with TD–TD pairs in the right inferior frontal gyrus (IFG) during joint attention tasks. A similar reduced IBS of ASD dyads was also found in the right temporal–parietal conjunction (TPJ) in more naturalistic contexts (Hirsch *et al*., 2022; Quiñones-Camacho *et al*., 2021; Du *et al*., 2024) (Fig. 4B, C). This brain region has been considered to play a vital role in the mentalizing and perspective-taking process, as its hyper-activation is consistently found when an individual tries to understand the mental states of other individuals (Filmer *et al*., 2019; Hyde *et al*., 2018). The reduced IBS of ASD dyads could be a result of the failure of communication between ASD individuals and their healthy partners. While the evidence of reduced IBS between ASD and experimenters was primarily established, the parent–child interaction study did not find significantly reduced IBS in the ASD group compared with the TD group (Kruppa *et al*., 2021).

### The association between IBS and phenotypes of ASD

Besides the reduced IBS strength of ASD dyads compared with TD dyads, the link between ASD clinical symptom profile and IBS could also serve as evidence supporting IBS as an effective measure of the social impairment in ASD. Specifically, previous studies have revealed the associations between IBS of ASD dyads and social communication-related scales score of ASD patients, such as the Social Responsiveness Scale-Second Edition (SRS-2) (Quiñones-Camacho *et al*., 2021), Autism-Spectrum Quotient (AQ) (Wang *et al*., 2020), and Autism Diagnosis Observation Schedule-Second Edition (ADOS-2) social effect domain and severity (Key *et al*., 2022). Wang *et al*. (2020) recorded the IBS between ASD children and their parents during a computer-based social cooperation task and a single-person task. They found that task-related parent–child IBS at the right prefrontal cortex negatively correlates with the total score of the AQ scale, AQ communication subscale, and AQ imagination subscale (Fig. 4A). By contrast, Du *et al*. (2024) found the participants’ performance during the theory of mind task, joint attention task, and imitation task were able to predict the IBS of the TD group instead of the ASD group. For adult ASD individuals, Quiñones-Camacho *et al*. (2021) found IBS during a free conversation task was negatively correlated with social communication impairments rather than restricted interests and repetitive behaviors measured by SRS-2. However, they only found a significant correlation on the whole sample, while no significant correlation was observed within either the ASD group or the TD group. In an EEG-based hyperscanning study, Key *et al*. (2022) also revealed a significant ASD symptom-related IBS pattern, where social interaction-induced alpha band IBS was negatively correlated with the Social Affect total scores of ADOS-2 in individuals with ASD.

In summary, with different modalities and data analysis methods, ASD symptom-related IBS has been observed in the bilateral TPJ, right prefrontal cortex, and IFG. However, the evidence regarding the difference between IBS of ASD dyads and TD dyads and the association between IBS and ASD-related symptoms was inconsistent and insufficient.

## Discussion

Extensive research has been invested in uncovering the neural underpinnings of social impairments in ASD, with the aim of enhancing therapeutic interventions for this complex neurodevelopmental condition. Despite this, conventional brain imaging modalities, including structural MRI and fMRI encounter challenges in pinpointing consistent biomarkers linked to naturalistic social interactions in ASD due to their stringent measurement constraints (Hernandez *et al*., 2015). Hyperscanning, a technique prominent in social cognitive neuroscience, offers substantial potential for elucidating the neural irregularities associated with social deficits in ASD (Babiloni and Astolfi, 2014; Zhao *et al*., 2024). In this review, we have synthesized and underscored the cutting-edge methodologies, experimental frameworks, and pivotal insights gleaned from hyperscanning research in ASD, providing a benchmark for future investigations. However, our review identified just eight studies employing hyperscanning to explore ASD-related IBS anomalies, underscoring an imperative for expanded research to harness the full potential of this approach. In the following sections, we will delineate the prevailing challenges associated with hyperscanning in ASD research and offer actionable recommendations to guide future studies in this domain.

### Addressing the inconsistent results of ASD-related IBS

In examining the limitations of previous research on the interaction between children with ASD and their parents, a notable inconsistency emerges in the findings regarding IBS. While studies involving adults with ASD have shown reduced IBS in dyads, five studies focusing on children did not yield consistent evidence linking atypical IBS to social impairments in ASD. For instance, Wang *et al*. (2020) reported a correlation between ASD symptom severity and the IBS of parent–child dyads; however, their reliance on the AQ rather than established clinical diagnostic tools such as ADOS-2 and SRS-2 raises questions about the validity of their conclusions. Furthermore, studies by Kruppa *et al*. (2021) and Tang *et al*. (2023) found no significant differences in IBS between ASD–parent and TD–parent dyads, despite noting behavioral synchrony variations. Overall, these findings suggest that the current body of research lacks robust evidence to definitively link ASD with atypical IBS, particularly in child–parent interactions.

The heterogeneity in divergent ASD-related IBS patterns observed across parent–child dyads may be attributed to several factors. First, the developmental nature of ASD inherently results in highly variable neural abnormalities across different ages before adulthood (Rommelse *et al*., 2017). Notably, the age range of children with ASD recruited in the reviewed studies varied substantially, spanning early childhood to adolescence. Excessive age heterogeneity within ASD groups probably contributed to greater intra-group variability in IBS patterns, potentially obscuring group-level differences between ASD and TD children. This interpretation aligns with the sole study that detected significant ASD–TD differences in IBS, which utilized a sample with minimal age variation (Du *et al*., 2024). Another group of critical factors is related to the diversity in parent–child interaction quality. While using naturalistic parent–child interactions enhances ecological validity compared to standardized partner paradigms, this approach fails to account for parenting style as a confounding variable, thereby complicating the interpretation of IBS findings (Crowell *et al*., 2019). It has been reported that parental stress significantly affected the behavioral outcomes of children with ASD (Clauser *et al*., 2021). These parenting-related variables could significantly influence the quality of parent–child interactions. To address these issues, future research should incorporate child age and gender as a covariate in statistical models and integrate behavioral assessments of parent–child interaction quality as moderators or covariates in analytical frameworks.

Besides the confounders of dyad-specific variables, the current hyperscanning methods have short backs that could be significantly advanced in future research. First, the existing fNIRS studies, though featuring high ecological validity, focused mainly on the frontal area and the temporal regions, such as the inferior parietal lobule (IPL) and TPJ (Du *et al*., 2024; Key *et al*., 2022; Kruppa *et al*., 2021; Wang *et al*., 2020). We argue that limited coverage of fNIRS channels may have hindered a more comprehensive understanding of IBS patterns in response to social interactions in ASD. Since the “social brain” network collaboratively supports social communication during human daily life in a coordinated way, the limited brain coverage might omit the interpersonal synchrony between brain networks that consist of multiple brain regions (Ashwin *et al*., 2007; Bilek *et al*., 2022). Promisingly, high-density fNIRS or diffuse optical tomography (DOT) systems have demonstrated enhanced spatial coverage and precise mapping of brain function, pushing its spatial resolution close to that of fMRI (Eggebrecht *et al*., 2012; Li *et al*., 2023). However, additional weight reduction and ergonomic issues with such systems remain to be addressed. We envision that in the coming years, portable fiberless high-density fNIRS devices (both tomography and topography designs) will be developed and optimized to reconcile the need for high spatial coverage and mapping in ASD hyperscanning investigation.

On the other hand, adopting a naturalistic paradigm that emphasizes face-to-face interactions is essential, moving away from keystroke tasks. Emerging evidence suggests that difficulties in processing social information from faces are a significant factor contributing to social impairments in ASD (Bookheimer *et al*., 2008; Corbett *et al*., 2009). Face-to-face interactions necessitate sustained interpretation of facial cues, which presents greater challenges than static images used in experimental settings. By creating a more naturalistic experimental environment, researchers can better assess the social communication abilities of children with varying levels of interaction skills.

### Exploration of the dynamic nature of IBS in social interactions

The time-varying IBS during social interactions is a critical aspect overlooked in all reviewed articles. Neural activity in the brain is inherently dynamic, both at rest and during social engagement (Alderson *et al*., 2018; B. Chen *et al*., 2023; Collins and Frank, 2018; Li *et al*., 2021). Although previous hyperscanning studies have employed various metrics to measure IBS using fNIRS, fMRI, and EEG, these analyses focus predominantly on the static characteristics of time series imaging data. In contrast, real-time social interactions often exhibit variable and complex participant behaviors, resulting in significant heterogeneity in their IBS patterns. Consequently, IBS during real-life social interactions is likely to fluctuate based on the social engagement of both members of the dyad.

Thus, it is essential to investigate time-varying IBS patterns with a more refined temporal resolution. Recently, there has been a growing interest in addressing this challenge through the exploration of dynamic IBS (dIBS) during social interactions (Li *et al*., 2021). This emerging research indicates that the dynamic characteristics of time-varying IBS states correlate with the interaction performance of dyads. By exploratively investigating the dIBS characteristics, such as variability, transition frequency, and state dwell time, we could identify the ASD-specific dIBS pattern and the associated domain of symptoms. Overall, compared to traditional static IBS measurements, dynamic IBS analysis may provide deeper insights into the neural mechanisms underlying real-life social interactions, particularly for individuals with ASD.

### Beyond IBS—multimodal measurement of inter-personal synchrony

IBS primarily measures neural coupling associated with advanced social functions, such as empathy, perspective-taking, and theory of mind in individuals with ASD. We propose that the physiological linkage between dyads—termed inter-personal synchrony—may reveal multi-dimensional synchronized activity during social interactions. Inter-personal synchrony extends beyond mere brain activity synchronization; it also encompasses physiological connections, known as physiological synchrony. This phenomenon can be quantified using various physiological measures, such as inter-beat-interval (IBI) and electrodermal activity (EDA). Emerging pilot studies have suggested that examining the physiological linkage between individuals with ASD and neurotypical counterparts could offer valuable insights into the social impairments associated with ASD (Baker *et al*., 2015; Dunsmore *et al*., 2019). Therefore, by integrating brain recording methods with physiological measurements such as IBI and EDA, we may gain a more comprehensive understanding of social impairments in ASD. Since physiological linkage reflects the synchrony between the fundamental emotions that are triggered in social interaction, it might promote the emergence of inter-brain synchrony. A recent study showed that higher mother–child IBS during competition than cooperation might be explained by their physiological synchrony (Reindl *et al*., 2022). Regarding the difficulty of children with ASD during emotion recognition (Corbett *et al*., 2009), we hypothesize that the association between physiological linkage and inter-brain synchrony found in TD individuals might be damaged in parent–autistic child dyads. We anticipate that inter-personal synchrony will facilitate a deeper exploration of the neural mechanisms underlying social behaviors and deficits in ASD. For individuals with ASD, who often display atypical patterns of brain activation and physiological responses during social interactions, these methods can help identify specific biomarkers or neural signatures associated with social difficulties, ultimately contributing to more effective interventions and support in their social lives.

### IBS as a potential ASD biomarker in the future

Hyperscanning-derived IBS shows significant promise as an objective neural biomarker for quantifying social impairment severity in ASD, with demonstrated correlations to established clinical metrics such as ADOS-2 and SRS-2 scores (Key *et al*., 2022b; Quiñones-Camacho *et al*., 2021). Its capacity to detect subtle social communication deficits in real-life social interactions makes IBS a potential tool for accurate diagnostics and personalized treatment. Further enrichment may arise from integrating IBS with physiological synchrony measures (e.g. IBI), creating multimodal biomarkers of social functioning that account for both neural and bodily coordination (Reindl *et al*., 2022). However, clinical translation could be hindered by methodological heterogeneity in experimental paradigms and IBS metrics, which impede cross-study standardization. Also, the generalizability of IBS as a biomarker is further limited by the potential confounders introduced by sample bias, including age, gender, and cultural background. Given that age and gender in autistic children have been reported as significant modifiers of neurodevelopmental abnormalities (Neuhaus *et al*., 2021), these factors should therefore be incorporated as covariates in future analyses. The influence of cultural contexts on IBS may manifest through differences in social impairment phenotypes among children with ASD across varying cultural backgrounds (de Leeuw *et al*., 2020; Perepa, 2014). Future studies should develop stable biomarkers within specific populations before proceeding to large-scale comparative studies across diverse demographic profiles. Finally, while other mental disorders, such as ADHD (attention-deficit hyperactivity disorder) and SAD (social anxiety disorder), also featured as a deficit of social communication, the disorder-specificity of IBS alterations does challenge diagnostic utility (Konrad *et al*., 2024; Yang *et al*., 2024). Advancing IBS toward clinical application necessitates standardized protocols for task design and synchrony metrics, coupled with rigorous validation through large-scale trials across diverse ASD subpopulations to establish reliability and specificity.

## Conclusion

While significant strides have been made in understanding the neural signatures of social deficits in ASD, much remains to be explored, particularly through advanced methods such as hyperscanning. The scarcity of studies employing hyperscanning to probe the IBS anomalies associated with ASD highlights an urgent need for more in-depth investigation in this domain. Addressing current methodological challenges, such as expanding fNIRS coverage and spatial resolution and employing more naturalistic paradigms, could significantly enhance the robustness and relevance of future findings. Additionally, the fusion of neuroimaging with physiological data presents a compelling strategy for unraveling the intricate dynamics of social engagement in ASD. By refining these methodologies and expanding research efforts, we can move closer to identifying reliable biomarkers for ASD social impairments, ultimately leading to more effective treatments and support for individuals with ASD in their social lives.
